# Mapping long‐range contacts between risk loci and target genes in human diseases with Capture Hi‐C

**DOI:** 10.1002/ctm2.183

**Published:** 2020-09-27

**Authors:** Canhui Cao, Qian Xu, Shitong Lin, Wenhua Zhi, Cordelle Lazare, Yifan Meng, Ping Wu, Peipei Gao, Kezhen Li, Juncheng Wei, Peng Wu, Guoliang Li

**Affiliations:** ^1^ Cancer Biology Research Center (Key Laboratory of the Ministry of Education) Tongji Hospital Tongji Medical College Huazhong University of Science and Technology Wuhan China; ^2^ Department of Gynecologic Oncology Tongji Hospital Tongji Medical College Huazhong University of Science and Technology Wuhan China; ^3^ National Key Laboratory of Crop Genetic Improvement Huazhong Agricultural University Wuhan China; ^4^ Agricultural Bioinformatics Key Laboratory of Hubei Province Hubei Engineering Technology Research Center of Agricultural Big Data 3D Genomics Research Center College of Informatics Huazhong Agricultural University Wuhan China

Dear Editor,

Ill‐shaped genome conformations contribute to gene dysregulation through long‐range chromatin contacts and are responsible for biological phenotypes of disease and carcinogenesis, particularly in breast cancer,^1^, ^2^ colorectal cancer,^3^, ^4^ and autoimmune diseases.^5^, ^6^, ^7^ Here, we review the identification of long‐range contacts between risk loci and putative target genes in the above diseases using Capture Hi‐C technology. We hope that this review will provide insights into the related molecular mechanisms via the three‐dimensional (3D) genome structure of these diseases.

Current research shows that over 95% of single‐nucleotide polymorphisms (SNPs) from genome‐wide association study (GWAS) are located in noncoding regions.^7^ However, the roles of these GWAS SNPs in human disease development are unclear. A large proportion of such GWAS SNPs have been identified as DNase I hypersensitive sites,^7^ which overlap with the binding sites of different transcription factors, such as CTCF, FOXA1, GATA3, P300, and ER‐α,^1^ or are enriched with active histone marks in different cell types with cell specificity^4^ (Figure 1A and B). Therefore, it is important to link risk loci with GWAS SNPs, as distal regulatory elements, to their target genes through long‐range chromatin interactions. Capture Hi‐C technology was developed to detect chromatin interactions between regions of interest (e.g., noncoding SNP regions as potential regulatory elements) and their target genes with reduced sequencing costs and improved read depth.^8^

**FIGURE 1:**
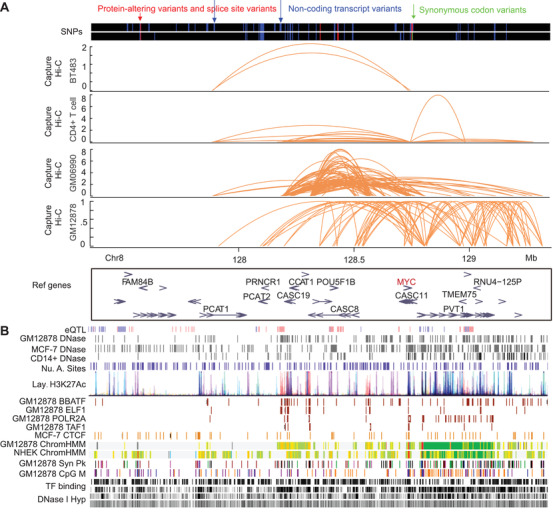
Capture Hi‐C was used to link noncoding risk loci to their target genes in human diseases through chromatin conformation for the underlying mechanisms and medical applications. A, SNPs and Capture Hi‐C results of BT483 cells, CD4+ T cells, GM06990, and GM12878 in 8q24.21 region (chr8:127,355,617‐129,355,617). B, Tracks of transcription factor binding sites, genes, eQTL, DNase I hypersensitive sites, ChIP‐Seq results, regulatory elements, and CpG methylation sites in 8q24.21 region (chr8:127,355,617‐129,355,617) from UCSC genome browser (hg19)

In breast cancer, 110 genes linked to 33 risk loci and seven genes linked to three risk loci have been identified using Capture Hi‐C technology, respectively^1^, ^2^ (Table S1). Many risk loci in these two studies had multiple target genes, and especially, 75% of these 36 risk loci had target genes other than their nearest genes. If the “nearest genes” annotation method was used, most target genes of these risk loci could not be found. In this sense, Capture Hi‐C is more effective in identifying the target genes of risk loci than the “nearest genes” annotation method. In their results, various common long‐range interactions have been identified, for example, 2q35(rs13387042) to IGFBP5, 8q24.21(rs13281615) to MYC and CCDC26, and 9q31.2(rs865686) to KLF4 (Figure 2A). Baxter et al also identified 62 interactions common in both ER+ and ER− cancer cells, as well as several interactions found in ER+ cells, but not in ER− cells, for example, rs2981579(FGFR2) and rs2236007(PAX9) in T‐47D,^1^ and the expression of them were high in T‐47D (Figure S1). Furthermore, some risk loci could interact with their target genes with a very long genomic distance. Baxter et al. found that there were much more interactions with distance above 2 000 kb in ER+ cancer cells than those in ER− cancer cells. With eQTL analysis, the association between SNPs and gene expressions can be identified. However, the molecular mechanism between SNPs and gene expressions is unclear. If there are long‐range chromatin interactions between SNPs and genes, such association could be explained partially. Baxter et al reported that the expression levels of MRPL34, COX11, and CDCA7 are associated with the 19p13.1 (rs8170), 17q22 (rs6504950), and 2q31.1 (rs1550623) genotypes, respectively; CTSW and SNX32 expression levels are associated with the 11q13.1 (rs3903072) genotype, and SSBP4 and LRRC25 expression levels are associated with the 19p13.11 (rs4808801) genotype^1^ (Figure S2). These genes were linked to the SNPs with chromatin interactions from Capture Hi‐C, and their expressions were associated with overall survival of patients in breast cancer (Figure S3).

**FIGURE 2:**
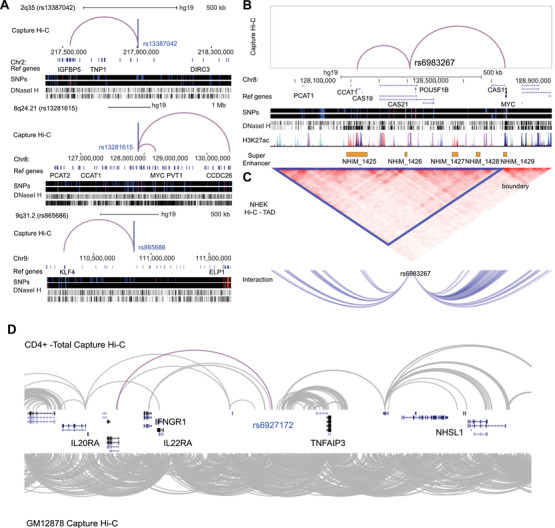
Capture Hi‐C was used to map the long‐range interactions between risk loci and target genes in human diseases. **A**, Capture Hi‐C interactions of 2q35 (rs13387042), 8q24.21 (rs13281615), and 9q31.2 (rs865686), which displayed three statuses: (1) bypassing the nearest genes, (2) with the nearest gene and the distant gene, and (3) with the nearest gene being the sole target gene. Chromosome range showed above, and genes were indicated in Ref genes. **B**, Capture Hi‐C results of 8q24.21 (rs6983267) in colorectal cancer. Regulatory elements were indicated below. **C**, TAD result and interaction peaks of rs6983267. **D,** Capture Hi‐C results of CD4+ cells and GM12878 in 6q23 region, red line showed the interaction between rs6927172 and IL20RA. The interaction peaks were downloaded from 3D Genome Browser^10^

In colorectal cancer, interactions with risk loci are often identified as *cis*‐interactions or trans‐interactions in chromatin conformations, with enrichment in regulatory elements.^3^, ^4^ For example, Jäger et al found that interaction anchors (9 kb resolution) of risk loci in HCT116 cells are enriched at regulatory elements, with 52.74% in enhancer regions and 32.96% in promoter regions, higher than the percentages of regulatory elements as enhancers and promoters in some normal cells, hepatocellular carcinoma, and chronic myelogenous leukemia^3^ (Table S2, Figure S4). Orlando et al found that 74% of interaction peaks in HT29 cells and 83% of interaction peaks in LoVo cells are within topologically associated domains (TADs).^4^ Jäger et al also reported that most interaction peaks of 8q24.21 (rs6983267)^3^ are within TADs (hg19/Chr8:128.20 Mb‐128.80 Mb) (Figure 2B and C), with increased regulatory interactions between rs6983267 and MYC and between rs6983267 and CCAT1 in the same TAD. Importantly, Orlando et al. also identified ETV1 as a target for single‐nucleotide variations (SNVs) in *cis*‐regulatory element regions in Chr.7:14 474 549‐14 477 471, with the SNVs associated with high expression of ETV1 and disease outcome in microsatellite stable colorectal cancer (MSS‐CRC)^4^ (Figure S5A, C, and E). RASL11A was also identified as a target for copy‐number variations (CNVs) in *cis*‐regulatory elements in Chr13:27.524 Mb‐27.529 Mb (Figure S5B), with the CNVs found to contribute to the high expression of RASL11A in colorectal cancer^4^ (Figure S5D).

In autoimmune diseases, common chromatin interactions in B and T cells have been identified, including rs1408272, rs610604, and rs911263^5^, ^7^ (Figure S6, Table S3). Borbala et al annotated chromatin interactions to gene promoters in B and CD34+ cells and found that interactions with promoters are enriched in loci associated with disease SNPs, especially autoimmune diseases^7^ (Figure S7, Tables S4‐S18). However, chromatin interactions in different cell types are diverse. For example, rs6927172 in the 6q23 region is correlated with a higher frequency of interactions with IL20RA and increased expression of IL20RA in CD4+ T cells, but not in CD19+ B cells.^9^

The above studies highlight current progress in Capture Hi‐C technology in the study of risk loci and their target genes, which can help clarify the mechanisms of pathological phenotypes in human diseases. Still, several limitations remain. First, the application of Capture Hi‐C is not universal due to technical restrictions and difficulties in library preparation. Second, the detection of SNPs based on GWAS analysis is insufficient to cover all risk loci for different diseases and the input loci are often incomplete. Based on current Capture Hi‐C practice, methods with a smaller number of cells should be developed for more human diseases, for example, single‐cell Capture Hi‐C, which would not only solve cell number limitations but also capture efficient long‐range interactions in different cell types.

## Supporting information


**Supplementary Figure 1: The expression of FGFR2 and PAX9 in GSE139670**. The expression of FGFR2(A) and PAX9 (B) in cells from GSE139670 dataset.
**Supplementary Figure 2: eQTL analysis**. eQTL analysis results in the mammary breast tissue of MRPL34, CTSW, SNX32, SSBP4, and LRRC25 from GTEx Portal (https://gtexportal.org/home/).
**Supplementary Figure 3: Survival analyses in breast cancer**. Overall survival analysis of MRPL34, COX11, CDCA7, CTSW, SNX32, SSBP4, and LRRC2 in breast cancer, the maps were downloaded from Kaplan‐Meier Plotter (https://kmplot.com/analysis/index.php?p=service&cancer=breast).
**Supplementary Figure 4: Annotations of interaction bins (9kb resolution) in different cells**. Annotations of interaction bins of the promoter (A) and enhancer (B).
**Supplementary Figure 5: Interactions between risk loci and target genes in colorectal cancer**. (**A**) Interactions between SNVs of cis‐regulation element regions and ETV1. (**B**) Interactions between CNVs of cis‐regulatory element regions and RASL11A. (**C**) EVT1 expression between groups with different variations. (**D**) RASL11A between CNV samples and wild type samples. (E) Survival meta‐analysis of EVT1 and RASL11A.
**Supplementary Figure 6: Capture Hi‐C interactions in total B cell and CD34 cells**. Capture Hi‐C results of rs1408272 (**A**), rs610604 (**B**), rs911263 (**C**). The interaction peaks were downloaded from 3D Genome Browser ^10^.
**Supplementary Figure 7: Interactions between promoters and GWAS SNP‐containing fragments**. GWAS SNP‐containing fragments in GM12878 (**A**) and CD34+ cells (**B**).

Supporting Information
