# Supplementary material for: Mapping long‐range contacts between risk loci and target genes in human diseases with Capture Hi‐C
Source: Clin Transl Med. 2020 Sep 27;10(5):e183. doi: 10.1002/ctm2.183 (PMC7520083; doi:10.1002/ctm2.183)

Supplementary Figure 1

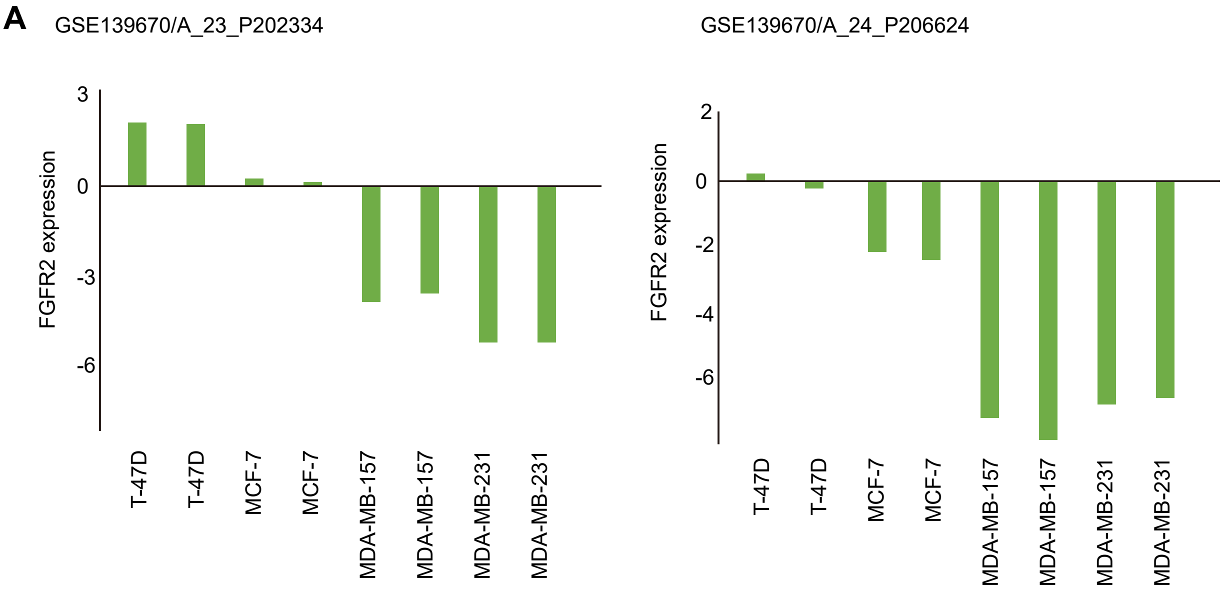

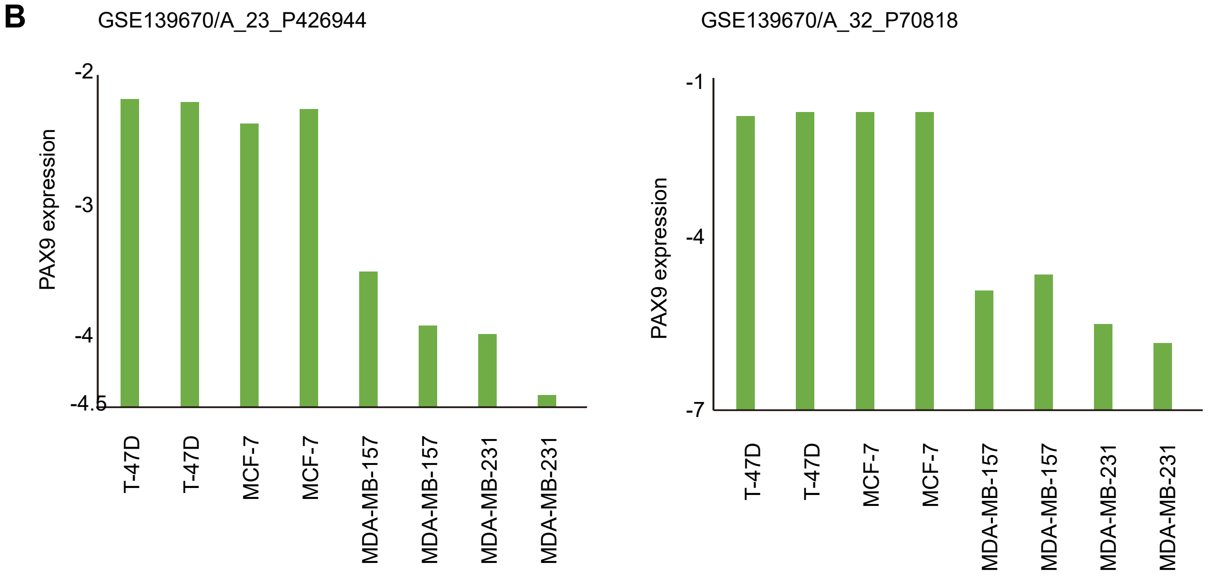

Supplementary Figure 2

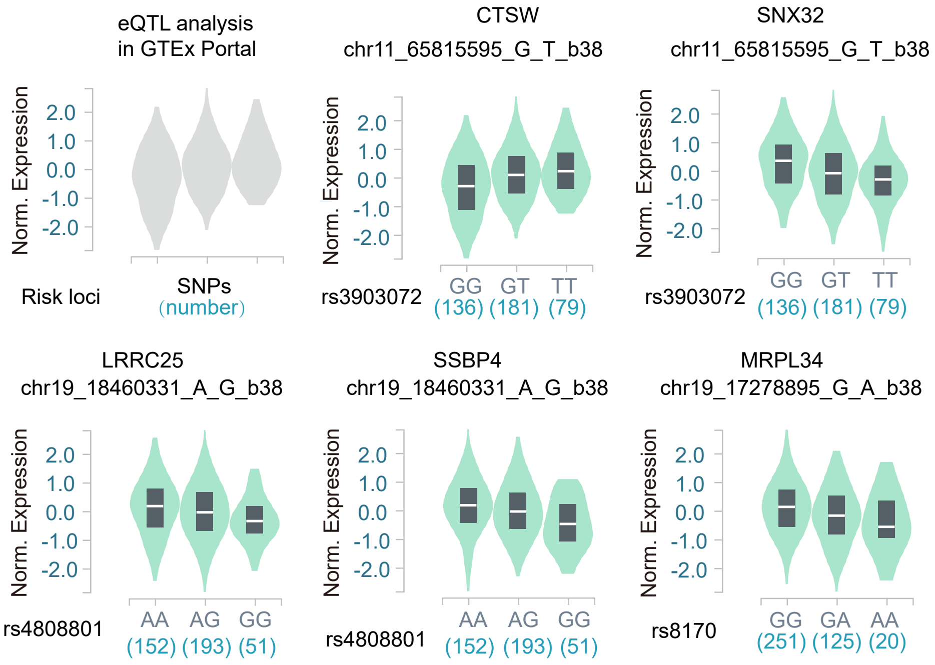

Supplementary Figure 3

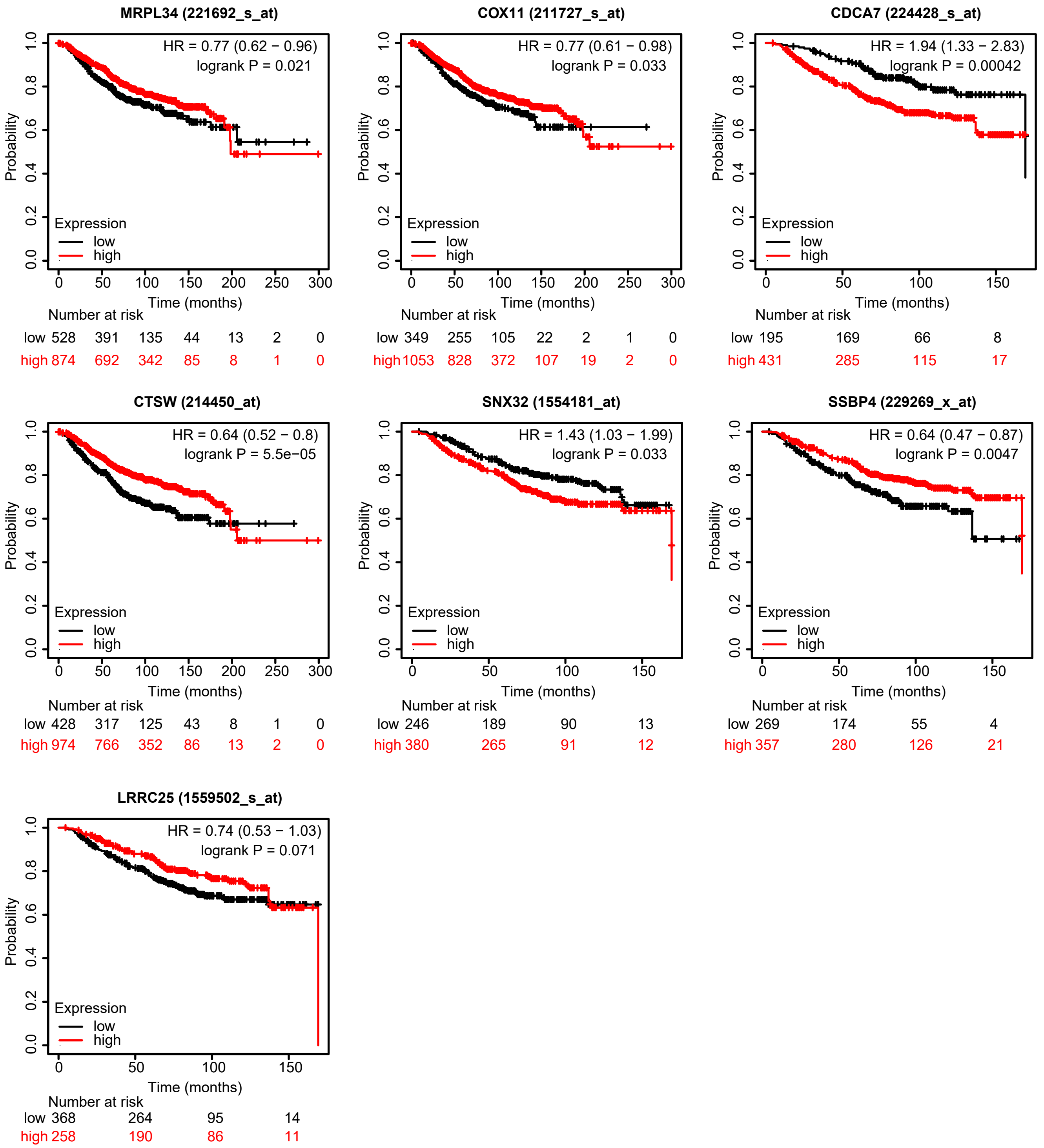

Supplementary Figure 4

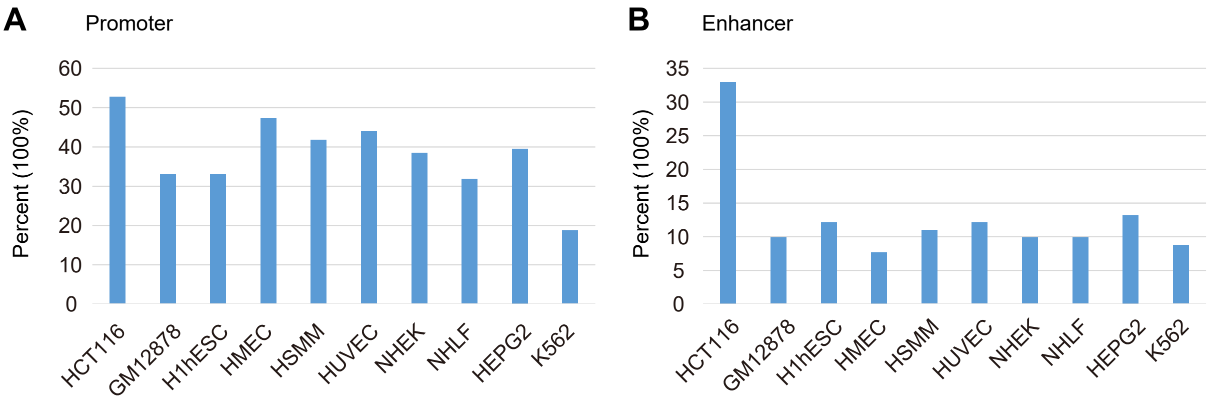

Data from reference 3

Supplementary Figure 5

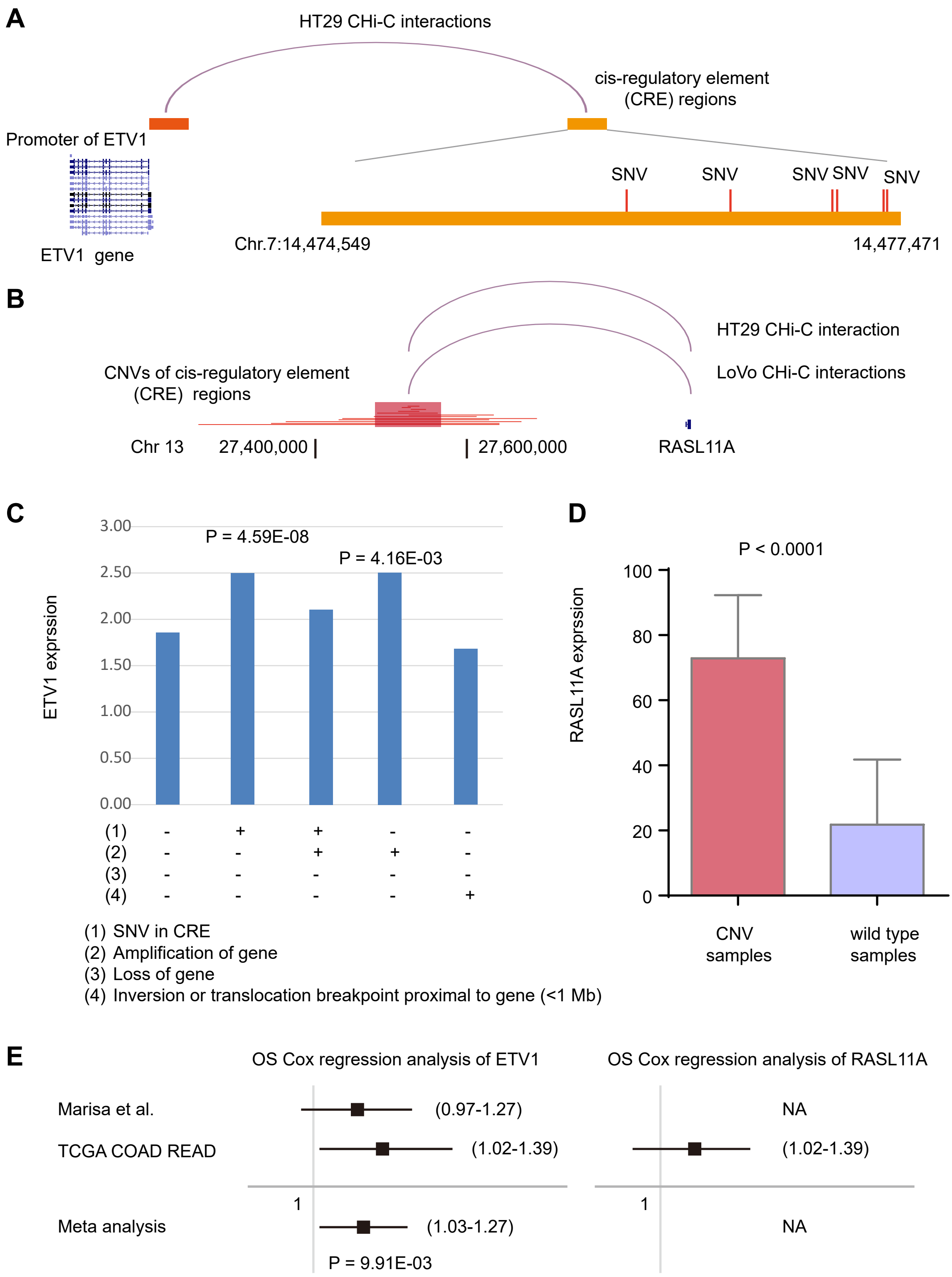

Supplementary Figure 6

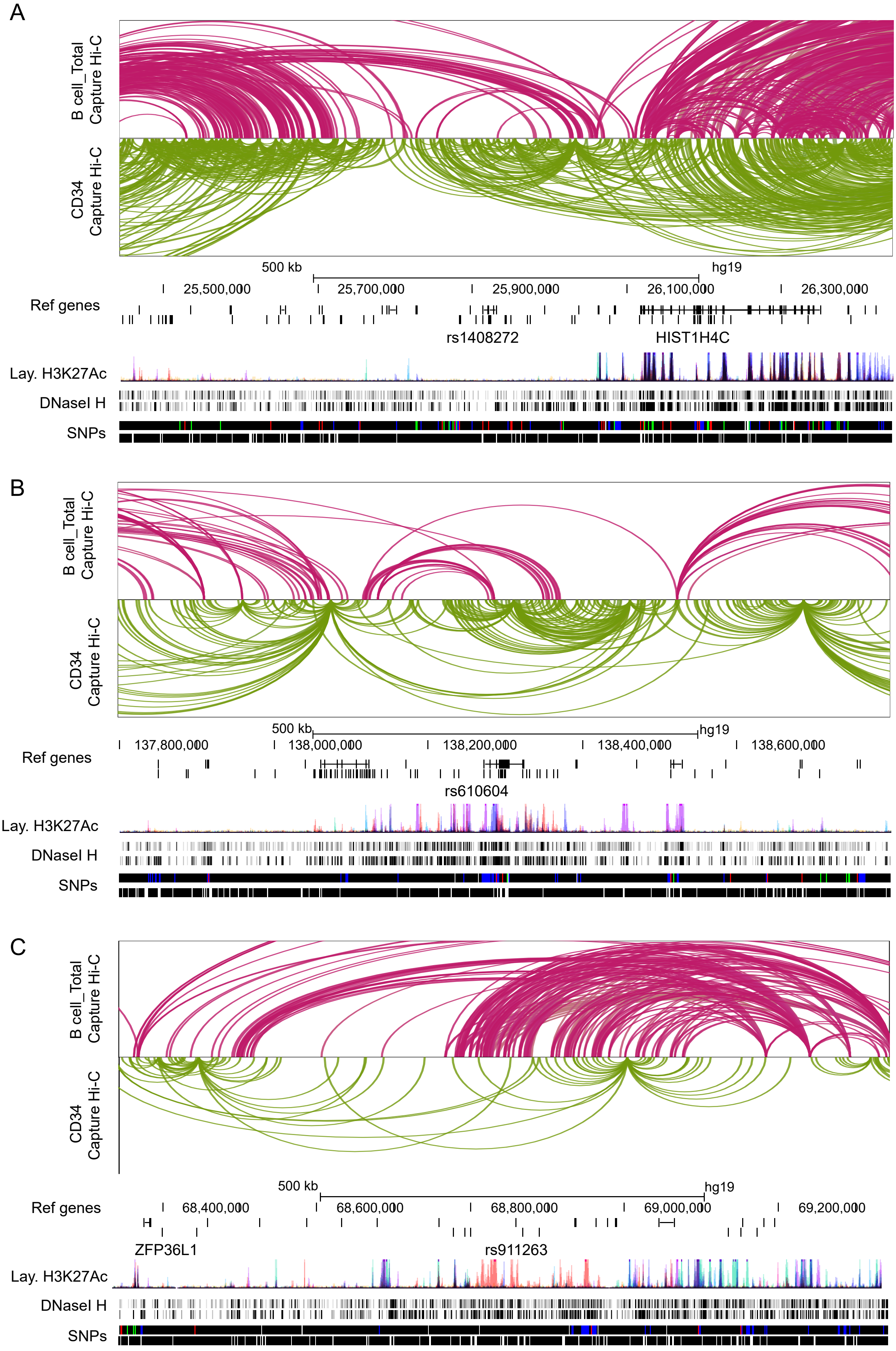

Supplementary Figure 7

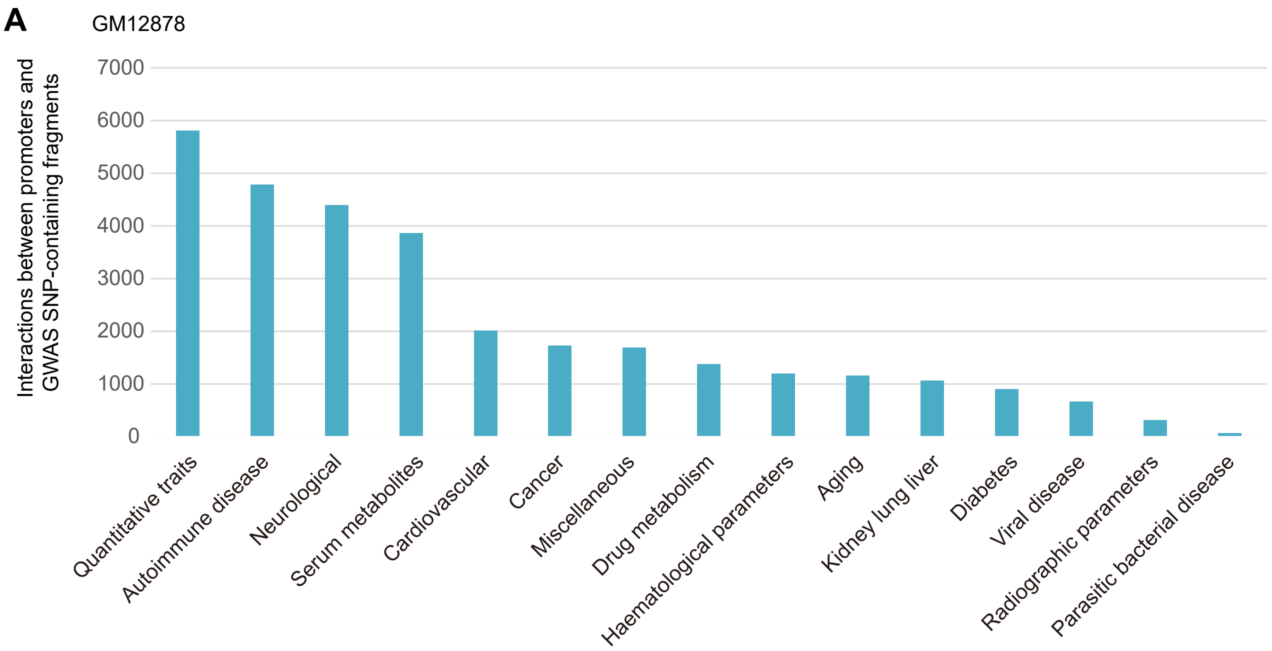

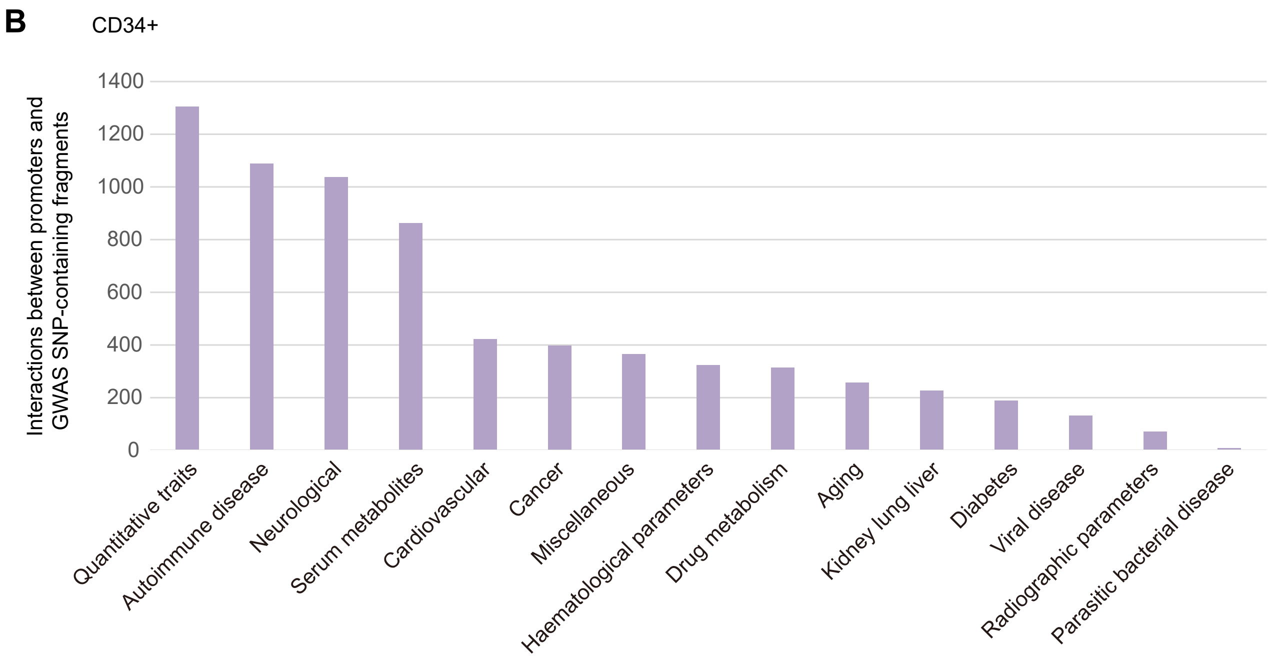

Supplement: Supplementary file 1 — Supplementary Figure 1: The expression of FGFR2 and PAX9 in GSE139670. The expression of FGFR2(A) and PAX9 (B) in cells from GSE139670 dataset. Supplementary Figure 2: eQTL analysis. eQTL analysis results in the mammary breast tissue of MRPL34, CTSW, SNX32, SSBP4, and LRRC25 from GTEx Portal (https://gtexportal.org/home/). Supplementary Figure 3: Survival analyses in breast cancer. Overall survival analysis of MRPL34, COX11, CDCA7, CTSW, SNX32, SSBP4, and LRRC2 in breast cancer, the maps were downloaded from Kaplan‐Meier Plotter (https://kmplot.com/analysis/index.php?p=service&cancer=breast). Supplementary Figure 4: Annotations of interaction bins (9kb resolution) in different cells. Annotations of interaction bins of the promoter (A) and enhancer (B). Supplementary Figure 5: Interactions between risk loci and target genes in colorectal cancer. (A) Interactions between SNVs of cis‐regulation element regions and ETV1. (B) Interactions between CNVs of cis‐regulatory element regions and RASL11A. (C) EVT1 expression between groups with different variations. (D) RASL11A between CNV samples and wild type samples. (E) Survival meta‐analysis of EVT1 and RASL11A. Supplementary Figure 6: Capture Hi‐C interactions in total B cell and CD34 cells. Capture Hi‐C results of rs1408272 (A), rs610604 (B), rs911263 (C). The interaction peaks were downloaded from 3D Genome Browser 10. Supplementary Figure 7: Interactions between promoters and GWAS SNP‐containing fragments. GWAS SNP‐containing fragments in GM12878 (A) and CD34+ cells (B). [file CTM2-10-e183-s001.pdf]
